# Healthcare provider perspectives on emergency department-initiated buprenorphine/naloxone: a qualitative study

**DOI:** 10.1186/s12913-023-10271-7

**Published:** 2024-02-15

**Authors:** Katherin Badke, Serena S. Small, Megan Pratt, Julie Lockington, Lara Gurney, Andrew Kestler, Jessica Moe

**Affiliations:** 1 Lower Mainland Pharmacy Services, Vancouver, BC Canada; 2 Pharmacy Department, Vancouver General Hospital, 899 W 12th avenue, Vancouver, BC V5Z 1M9 Canada; 3 Faculty of Pharmaceutical Sciences, University of British Columbia, Vancouver, BC Canada; 4 Department of Emergency Medicine, University of British Columbia, Vancouver, BC Canada; 5 Centre for Clinical Epidemiology and Evaluation, Vancouver Coastal Health Research Institute, Vancouver, BC Canada; 6 Social Work Department, Vancouver General Hospital, Vancouver, BC Canada; 7 Department of Emergency Medicine, Vancouver General Hospital, Vancouver, BC Canada; 8 School of Nursing, University of British Columbia, Vancouver, BC Canada; 9 Department of Emergency Medicine, St. Paul’s Hospital, Vancouver, BC Canada; 10 Department of Emergency Medicine, BC Children’s Hospital, Vancouver, BC Canada; 11 BC Centre for Disease Control, Vancouver, BC Canada

**Keywords:** Attitude of Health Personnel, Canada, Emergency Service, Hospital, Buprenorphine, Naloxone Drug Combination, Therapeutic use, Opiate Substitution Treatment, Opioid-Related Disorders, Drug therapy, Emergency Nursing, Pharmaceutical Services

## Abstract

**Background:**

Take-home buprenorphine/naloxone is an effective method of initiating opioid agonist therapy in the Emergency Department (ED) that requires ED healthcare worker buy-in for large-scale implementation. We aimed to investigate healthcare workers perceptions of ED take-home buprenorphine/naloxone, as well as barriers and facilitators from an ED healthcare worker perspective.

**Methods:**

In the context of a take-home buprenorphine/naloxone feasibility study at a tertiary care teaching hospital we conducted a descriptive qualitative study. We conducted one-on-one in person or telephone interviews and focus groups with ED healthcare workers who cared for patients given take-home buprenorphine/naloxone in the feasibility study at Vancouver General Hospital from July 2019 to March 2020. We conducted 37 healthcare worker interviews from December 2019 to July 2020. We audio recorded interviews and focus groups and transcribed them verbatim. We completed interviews until we reached thematic saturation.

**Data analysis:**

We inductively coded a sample of transcripts to generate a provisional coding structure and to identify emerging themes, which were reviewed by our multidisciplinary team. We then used the final coding structure to analyze the transcripts. We present our findings descriptively.

**Results:**

Participants identified a number of context-specific facilitators and barriers to take-home buprenorphine/naloxone provision in the ED. Participants highlighted ED conditions having either facilitative or prohibitive effects: provision of buprenorphine/naloxone was feasible when ED volume was low and space was available but became less so as ED volume increased and space decreased. Similarly, participants noted that patient-related factors could have a facilitative or prohibitive effect, such as willingness to wait (willing to stay in the ED for study-related activities and buprenorphine/naloxone initiation activities), receptiveness to buprenorphine/naloxone, and comprehension of the instructions. As for staff-related factors, time was identified as a consistent barrier. Time included time available and time required to initiate buprenorphine/naloxone (including time building rapport). Healthcare worker familiarity with buprenorphine/naloxone was noted as either a facilitating factor or a barrier, and healthcare workers indicated that ongoing training would have been advantageous. Many healthcare workers identified that the ED is an important first point of contact for the target patient population.

**Conclusion:**

Integrating a buprenorphine/naloxone program into ED care requires organizational supports (e.g., for managing buprenorphine/naloxone within limitations of ED volume, space, and time), and ongoing education of healthcare workers to minimize identified barriers.

**Supplementary Information:**

The online version contains supplementary material available at 10.1186/s12913-023-10271-7.

## Introduction

In Canada, there were 38,514 apparent opioid toxicity deaths between January 2016 and March 2023, and overdose deaths have increased since the COVID pandemic began [1]. Public health data from British Columbia (BC) shows that 60% of individuals with an overdose had at least one emergency department (ED) visit in the 12 months prior to the overdose, indicating that the ED is a vital point of contact for this patient population [2].

Buprenorphine/naloxone is a first-line treatment for opioid use disorder (OUD), and has been shown to decrease mortality [3, 4]. In a randomized trial in the United States, ED initiation of buprenorphine/naloxone led to 78% retention in addictions treatment at 30 days, compared to 37% in the group with referral to addictions care alone, however the effect waned by six months [5, 6]. Compared to methadone, buprenorphine/naloxone is safer for take-home dosing, even during the initiation period, and takes less time to achieve a therapeutic dose [3].

We conducted this qualitative study in the context of a larger feasibility study, initiated in July 2019, in which our multidisciplinary team implemented a new program providing take-home buprenorphine/naloxone packages to eligible ED patients [7]. In previous research on providing take-home naloxone kits from the ED, physicians identified lack of knowledge, time, training, and institutional support as barriers to the intervention [8]. Additionally, known barriers to community and inpatient initiation of opioid agonist therapy identified by healthcare providers include stigma (e.g., negative views of individuals with OUD or OUD treatment) [9–14], logistics (e.g., time or space limitations, lack of access to allied health support, lack of access to specialists, and lack of access to follow up) [9–15], and provider knowledge of OUD medications (e.g., insufficient training, or lack of confidence regarding knowledge of OUD treatment) [9–11, 13–15]. To address and minimize these barriers prior to initiating the take-home buprenorphine/naloxone program at our site, our team implemented a novel, multidisciplinary approach to ED buprenorphine/provision that included a comprehensive education plan for ED healthcare workers and a qualitative evaluation plan that thoughtfully engaged each healthcare worker group. Prior to this study there was no formal program for starting buprenorphine/naloxone from this ED and ED patients were not screened systematically for OUD.

The evaluation plan involved conducting qualitative interviews with the emergency physicians and focus groups with the nurses, pharmacists, and social workers using an interview guide created by the multidisciplinary research team. The primary objectives of this qualitative study were to understand ED healthcare worker perceptions of an ED’s role in providing take-home buprenorphine/naloxone within our unique multidisciplinary program, and to characterize healthcare worker identified facilitators and barriers to take-home buprenorphine/naloxone provision in the ED.

## Methods

### Study design, setting, and time period

We undertook a descriptive, qualitative study using semi-structured focus groups and interviews. We conducted one-on-one in person or telephone interviews and focus groups with ED healthcare workers who cared for patients given take-home buprenorphine/naloxone in a feasibility study at Vancouver General Hospital, a tertiary care hospital in Vancouver, Canada with an annual census of 95,000 ED visits. We enrolled patients in the feasibility study between July 2019 to March 2020, and we completed interviews and focus groups with healthcare workers between December 2019 and July 2020. Based on barriers identified in prior research in non-ED healthcare settings [9–13, 15], we provided multidisciplinary training specific to each healthcare worker group, study team logistical support, and divided the responsibilities for take-home buprenorphine/naloxone initiation across the ED healthcare team. We engaged people with lived and living experience of opioid use throughout study design and implementation.

### Feasibility study enrollment

The study assessed the feasibility of randomization to standard dose or microdose buprenorphine/naloxone. Research assistants screened patients in the ED based on chief complaint, then approached them to inquire about non-medical opioid use and OUD. Staff members in the ED could also identify potential patients. Emergency physicians evaluated patients for appropriateness of buprenorphine/naloxone. Pharmacists were primarily responsible for counselling patients, with delegated responsibility to physicians if a pharmacist was not available to counsel. Enrolled patients were eligible to receive a social worker consult if the patient was agreeable, and a social worker was available. Based on data from 14 patients in our feasibility study, mean time to consent patients was 14 minutes (SD 10), to counsel was 12 minutes (SD 4), and to complete an enrollment from first contact with the patient was 172 minutes (SD 104).

### Feasibility study intervention

Our standard dosing regimen included a three-day take-home package, starting at a dose of buprenorphine/naloxone 2 mg/0.5 mg, which could be repeated hourly to a maximum of 12 mg/3 mg in 24 hours. For days two and three, patients took the total dose achieved on day one (target 12/3 mg). Our microdosing regimen included a six-day take-home buprenorphine/naloxone package with an initial dose of 0.5 mg/0.125 mg twice daily, which increased gradually to 12 mg/3 mg daily by day six. Our induction protocols have been published elsewhere and additional information is available in Appendix I [7].

### Counselling and timing of first dose

Counselling included discussing buprenorphine/naloxone indication, mechanism, recommendation for time of initiation, adverse effects, over-the-counter options for managing withdrawal symptoms, and reviewing an educational pamphlet on buprenorphine/naloxone. Patients were discharged with take-home packages to initiate in the community. For standard dosing, the patient needed to be abstinent from opioid use and wait until they were in moderate to severe withdrawal following discharge from the ED prior to initiation. If the patient was already in moderate to severe withdrawal at time of presentation, they were offered a standard dosing induction while in the ED. For microdosing they could initiate at any time that was convenient to them, including in the ED, if preferred. Due to ED space constraints, patients could be in private rooms, private curtained areas, hallway stretchers, or hallway chairs depending on their chief complaint. When in hallways, every effort was made to find a private, confidential space for counselling. These constraints are similar for ED patients with other presenting concerns (e.g., mental health presentations).

### Follow-up

We referred patients to rapid access, low-barrier addictions clinics (open seven days a week) available in our setting to follow-up for ongoing opioid agonist therapy. The Vancouver Coastal Health overdose outreach team, who provide service navigation and linkage to health and social services including substance use treatment for people who have recently experienced an opioid overdose and/or are at high risk for opioid overdose, also attempted to follow up with all patients who lived in their catchment area [16].

### Costs to patient

There were no costs to the patient for the ED visit, initial buprenorphine/naloxone kits provided from the ED, or follow-up clinics, which were covered under a publicly funded healthcare system. Ongoing coverage for buprenorphine/naloxone or other opioid agonist therapies were covered under a government program, which is based on an individual’s income [17].

### Qualitative study methods

We planned to conduct 15 to 30 minute interviews with the emergency physicians and one to two hour focus groups with the nurses, pharmacists, and social workers. We opted for individual interviews with the physicians due to difficulty coordinating multiple physicians’ schedules, whereas focus groups were optimal for the other provider groups because we could coordinate these for multiple individuals at a time who were on similar shift rotations. Our facilitator followed a semi-structured guide for both interviews and focus groups (Appendix 2). We conducted initial interviews and focus groups at an on-site research office and transitioned to telephone-based interviews for all healthcare workers from March 2020 onwards. We approached this study through a social constructionist epistemology, acknowledging the co-creation of knowledge between researchers and subjects [18]. We obtained ethics approval from the University of British Columbia Clinical Research Ethics Board (H19-00889). We present the study results in accordance with the COREQ guidelines (Appendix 3). All participants gave informed verbal consent to participate in the study. Additional information on methods is available in Appendix 4.

### Recruitment

Multidisciplinary healthcare workers who provided care to patients enrolled in our ED feasibility study, including physicians, registered nurses, pharmacists, and social workers were eligible to participate in this qualitative study. Upon patient enrollment, research assistants recorded the e-mail addresses of the healthcare providers involved in the care of patients. We excluded healthcare workers if they were involved in study planning or execution, or if the study team had no valid contact information for them*.* We invited eligible healthcare workers via e-mail to participate in a focus group or interview, first in-person, and then via telephone due to the COVID-19 pandemic. We sent two reminder emails to those who did not respond.

### Data collection

We developed a semi-structured discussion guide that addressed themes identified in the literature. The principal investigators (KB, JM) created the initial drafts, and other members of the multidisciplinary research team (JL, LG, MP, SSS) revised and edited the discussion guides prior to data collection. During sessions, we allowed participants to engage in open dialogue and to discuss new concepts.

Most study team members were practicing clinicians (physicians, pharmacist, nurses, and a social worker), so a research coordinator trained in qualitative methods (SSS) external to the healthcare team and with no pre-existing relationship to participants conducted all interviews and focus groups, which protected participant confidentiality and reduced potential response bias due to pre-existing relationships between participants and clinical study team members. The qualitative researcher led the interviews and focus groups over the phone or at a research office on-site. She explained the study goals and objectives and obtained verbal consent at the beginning of each interview or focus group. We completed interviews and focus groups separately among physicians, nurses, and pharmacists until no novel concepts or themes began to emerge through interviews, at which time we felt we had reached data saturation. Due to the small social worker sample size, we conducted interviews until there were no additional consenting participants. Seven social workers cared for patients in the feasibility study, one of whom was excluded as they were part of the research team. Of the remaining social workers, four consented to participate.

Initially, we conducted interviews and focus groups in person at a research office in Vancouver, but we shifted to telephone-based one–on-one interviews due to restrictions introduced during the COVID-19 pandemic. We completed all physician interviews and two nurse focus groups in person prior to the start of the COVID-19 pandemic, after which we conducted all remaining interviews one-on-one over the phone. The healthcare interviews and focus groups lasted 14 to 120 min. Interviews and focus groups were supplemented with field notes and audio recorded. Research assistants and a professional transcriptionist then subsequently transcribed audio recordings verbatim for analysis. Transcripts were not returned to participants for comment.

### Data analysis

We coded and analyzed transcriptions using NVivo 12 (QSR International, version 12, 2020). We created a provisional coding framework based on the discussion guide and applied the provisional coding framework to a random sample of transcripts for each participant group, including representative subsets by clinician type. We met to discuss emerging themes following preliminary coding and to revise the coding framework. Subsequently, the qualitative researcher (SSS) iteratively coded and analyzed the data using a descriptive approach, applying the revised coding frame to all transcripts, which organized participant comments along the following themes: familiarity with and perceptions about buprenorphine/naloxone; experience starting patients on buprenorphine/naloxone; and facilitators and barriers to starting patients on buprenorphine/naloxone.

## Results

**Fig. 1:**
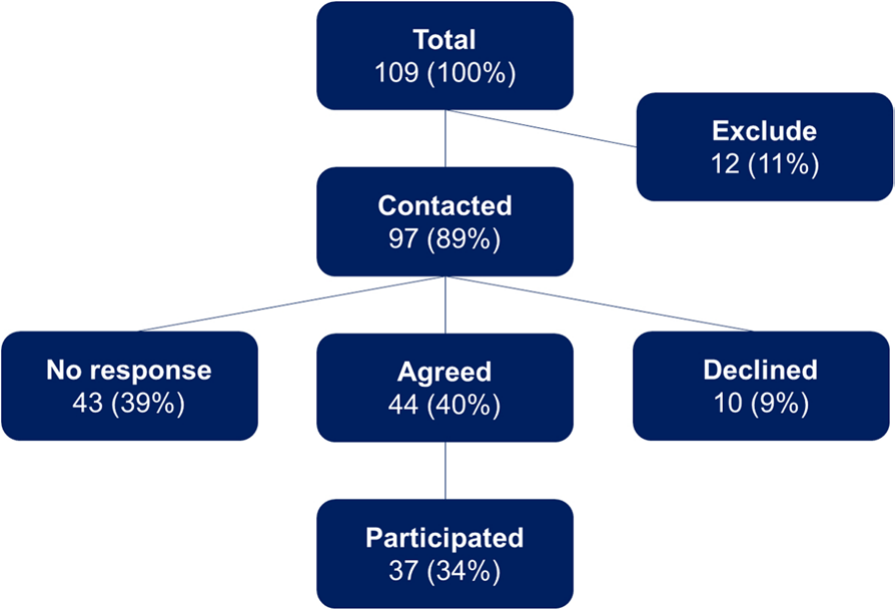
Healthcare worker eligibility and participation

We enrolled 68 patients in the feasibility study, of whom eight were excluded post enrollment, 14 left the ED against medical advice, 21 received standard dosing, and 25 received microdosing [7]. In total, 109 ED healthcare workers (37 physician, 47 nurses, 18 pharmacists, and seven social workers) who provided care for patients in the feasibility study were eligible for this study. Twelve healthcare workers were excluded: five because they were involved in study planning or execution and seven because the study team had no valid contact information for them. We contacted 97 healthcare workers, of whom 44 agreed to participate. We interviewed 37 clinicians (ten physicians, fourteen nurses, nine pharmacists, and four social workers) between December 2019 and July 2020 (Fig. 1).

### Healthcare worker characteristics

Two healthcare workers had worked in their field for less than one year, 15 had worked in their field for one to four years, 11 had been in their field for five to nine years, and nine had been working for ten or more years.

### Baseline knowledge

Many (21/37, 57%) of the workers reported at least some exposure to buprenorphine/naloxone through a previous patient encounter, or previous role or job. The remainder reported being aware of buprenorphine/naloxone through education or other training prior to the study (10/37, 27%) or having no experience with buprenorphine/naloxone prior to the study (6/37, 16%).

### Role of ED in starting buprenorphine/naloxone

Many healthcare workers (26/37, 70%) identified that the ED is an important first point of contact for the target patient population. All healthcare workers agreed with the idea of initiating buprenorphine/naloxone in the ED.

Physician 010 *“It’s evidence-based, first line therapy for opioid use disorder and so I think that all patients with opioid use disorder or identified as such when they present to the department should be at least offered therapy in the same way we would offer antibiotics for an infection.”*

Pharmacist 107 *“…a lot of patients … would be like ‘oh, where do we even start’ or ‘where do we even go?’ I mean, my sense is that there’s a lot of resources out there, but if you have to kind of pinpoint where to start, it feels like it is something that might be very difficult… Whereas emerg, it’s like ‘oh ok’ everyone knows the emerg. It’s…a safe starting point….”*

Social Worker 301 *“It gave me faith back in the department or in the role of addiction treatment in the department because typically I find that those patients that we see who are ready for change or are wanting some help for their addiction, it doesn’t come easy in the emergency department.”*

Nurse 203 “*I’m just there to throw the ball out. It’s the patient’s decision, what they want to do with it, with everything. You know, like, every time you come in, I’m going to ask you these questions because maybe one day you will take it on board…”.*

### ED-specific facilitators and barriers (Table 1)

**Table 1:** Healthcare workers’ perceptions of ED factors as facilitators and barriers

Factor	Participant	Facilitator	Barrier
Patient volumes in the ED, competing priorities	*19/37 (51%)* *(10 nurses, 3 pharmacists, 5 physicians, 1 social worker)*	*So, one patient, and this was the patient who had never been on Suboxone [buprenorphine/naloxone] before, and … was an overnight patient. But our department was not busy, and I believe he probably came in around three or four in the morning and he was willing to wait ‘til the morning. (Physician 010)*	*…there’s people that are sicker and require more intervention and more attention. (Nurse 201)* *…there are so many patients in emerg and there are so many things that … I’m doing. (Pharmacist 105)*
Availability of space	10/37 (27%) (4 nurses, 2 pharmacists, 4 physicians)	*…she had already had … an appropriate bed (Pharmacist 108)* *…our department was not busy … There was no need for space. (Physician 010)*	*So they were in the hallway in a chair, which made it kind of hard to communicate in a way that was really supportive regarding Suboxone [buprenorphine/naloxone]… it really wasn’t an ideal location to be sitting and...having this confidential conversation.* (Nurse 213)
Time required & time available	6/37 (16%) (3 nurses, 1 pharmacist, 2 social workers)	*[None]*	*…those issues are usually quite large and in-depth … But we often don’t have the time because it’s like 'oh, now I have to go attend to a cardiac arrest, so see you later.’ (Nurse 205)* *I think, the trick with these—this patient population, is you got to really spend the time building rapport and relationship with them to be able to, you know, successfully introduce these ideas. (Social worker 301)*
Environment or atmosphere	3/37 (8%) (1 nurse, 1 physician, 1 social worker)	*So yes, you’re [in] an emergency department. You’re in a safe environment, you’re getting your needs met, whether the medication, food… (Social Worker 304)*	*…. the emergency department isn’t an appropriate place to do a regular [standard] induction. It’s chaotic, it’s loud, it’s bright, it stinks… sometimes, and … you’re nauseous, you’re having diarrhea, you don’t have the access to a toilet. (Nurse 201)*

Among healthcare workers, patient volume was frequently cited as having an impact on take-home buprenorphine/naloxone provision in the ED. High patient volume led to competing priorities for healthcare workers, limited available patient spaces, and affected healthcare workers’ ability to spend the necessary time with patients. In rare instances of low patient volume, healthcare workers reported having fewer competing priorities, easier access to a confidential space, and more time.

### Perception of patient-related facilitators and barriers (Table 2)

**Table 2:** Healthcare Workers’ perception of patient-related factors as facilitators and barriers

Factor	Participant Profile	Facilitator	Barrier
Patient willingness to wait / stay in ED	20/37 (54%) (5 nurses, 3 social workers, 7 pharmacists, 5 physicians)	*…luckily the patient was willing to stay….* (Physician 004) *…I had a patient who seemed pretty motivated to wait around for the study* (Pharmacist 108) *…. there was also some concern that this guy would, … leave prior to everything being sorted out…he stuck around, so that was good.* (Nurse 213)	*…sometimes in the process of trying to enroll them they might just leave the hospital. So I think that's happened once before. And just trying to get all the ducks aligned before you’re done and the patient has already left.* (Pharmacist 107) *Often, when they are awake and ready to go and also getting the message from the medical team … ‘Get out of here,’ they don’t wait to see the social worker*. (Social Worker 303)
Healthcare worker perceptions of a patient’s receptiveness, disposition, cooperation	20/37 (54%) (7 nurses, 3 social workers, 4 pharmacists, 6 physicians)	*…as long as they're receptive and … open to trying it, normally that process is very easy…* (Nurse 210) *…you must be potentially receptive to doing this* (Social Worker 304) *….what made it easy was that the patient wanted it.* (Physician 004)	*It was a bit of a challenge to kind of engage with them. They might not have necessarily wanted to listen* (Pharmacist 107) *Patients …didn’t want Suboxone* *[buprenorphine/naloxone]* *therapy. They didn’t want to mess around with things or go through sort of the withdrawal experience to get onto Suboxone [buprenorphine/naloxone]…* (Nurse 214)
Drowsiness, level of consciousness	10/37 (27%) (4 nurses, 4 pharmacists, 2 physicians)		*…the patient was … quite drowsy because she used opioids that day and so the pharmacist didn’t feel they were able to consent the patient properly at that time or counsel the patient properly.* (Physician 008) *I had maybe two patients who were really drowsy, and in those cases you really had to wake them up … and if they weren’t you know as awake then I would wonder if they got all of it* (Pharmacist 109)
Comprehension, retention of information	8/37 (22%) (3 nurses, 5 pharmacists)	*….she had already been on Suboxone* *[buprenorphine/naloxone*] *therapy before so the counselling was much shorter.* (Pharmacist 105)	*… educating could be a little bit difficult if they’re currently on drugs while they’re in the ED, how much information gets retained, how productive is the conversation.* (Nurse 206)

Healthcare workers also cited patient-specific facilitators and barriers to providing take-home buprenorphine/naloxone in the ED. The primary factors cited were patients’ willingness to stay in the ED for study-related activities and for buprenorphine/naloxone initiation activities (e.g. counselling), their degree of receptiveness, level of consciousness, and comprehension of information. Patients’ previous experience with buprenorphine/naloxone was generally a facilitator, as counseling was faster. Pharmacists were more likely to be concerned with patient comprehension and retention of information, possibly due to their greater role in medication counseling. Patient comprehension was a concern in some cases due to patient alertness (e.g., due to recent drug use) or limited understanding of the complexity of the buprenorphine/naloxone regimen (counselling was found to be easier for patients who had prior knowledge of buprenorphine/naloxone). Three social workers mentioned that patients’ fear of discrimination is a barrier to take-home buprenorphine/naloxone provision. Some healthcare workers (9/37, 24%) also mentioned psychosocial factors outside of the ED (e.g., no fixed address) as barriers to take-home buprenorphine/naloxone provision.

### Healthcare worker related facilitators and barriers

About half of the healthcare workers (19/37, 51%) noted that healthcare workers’ familiarity with buprenorphine/naloxone could facilitate or hinder the study process. Healthcare workers indicated that ongoing training would have been advantageous, which is consistent with other studies. The majority of healthcare workers (27/37, 73%) stated that research assistant support was a facilitating factor. Many healthcare workers (26/37, 70%) also noted that study materials, packaging, and training were facilitators.

Physicians were most likely to report a positive effect of collaboration with other staff (6/10, 60% physicians). Physicians noted that pharmacists played an important role in these processes, primarily by providing patient counseling, and also recognized the importance of support from nurses and social workers. Some healthcare workers (5/37, 14%) suggested that a specialized addictions healthcare worker would improve ED take-home buprenorphine/naloxone provision (e.g., nurse, pharmacist, or social worker). Healthcare workers identified that clear follow-up instructions on take-home buprenorphine/naloxone packages were beneficial, yet some still had concerns about ease of access to follow-up pathways after patients left the ED. Those who provided take-home buprenorphine/naloxone multiple times found that the burden decreased as they repeated the process with subsequent patients.

## Discussion

### Interpretation of findings

To our knowledge, ours is the first study to specifically elicit interdisciplinary healthcare workers perspectives toward ED initiation of buprenorphine/naloxone among separate healthcare worker groups, including clinical pharmacists, who were directly involved in patient care. A great majority of participants identified that the ED was a crucial location to offer buprenorphine/naloxone, given that it is a unique point of contact with individuals who may not be seen elsewhere in the healthcare system. Participants in this study highlighted several ED conditions as having either facilitative or prohibitive effects on the provision of buprenorphine/naloxone. Importantly, buprenorphine/naloxone provision was feasible when ED volume was low, and space was available but became less so as ED volume increased and space decreased. When the ED volume was higher this did make it more challenging to access a private care space for counselling, which is a similar issue experienced in the ED for other confidential conversations with patients. Similarly, participants noted that patient-related factors could have a facilitative or prohibitive effect, such as willingness to wait for study-related activities and buprenorphine/naloxone initiation activities, receptiveness to buprenorphine/naloxone, and overall comprehension. Time was a consistent barrier, regardless of related ED or patient factors. Time to identify patients with substance use disorder, time to assess if a patient was appropriate for buprenorphine/naloxone, time to establish rapport, and time to counsel a patient were all factors contributing to time constraints. Participants highlighted the importance of training and interdisciplinary collaboration as having net positive effects on their perceived ability to counsel patients on take-home buprenorphine/naloxone. Although not an ED-related factor, follow up pathways were mentioned in the interviews, and were necessary to support the success of our ED take-home buprenorphine naloxone program. Rapid access, low-barrier addictions clinics are available in our setting and we referred patients to these to follow-up for ongoing opioid agonist therapy prescriptions. Our findings support that a multidisciplinary approach may improve patient access to ED buprenorphine/naloxone, and that specifically trained ED pharmacists may expand the capacity of EDs to offer this intervention.

### Comparison to previous studies

Consistent with our findings, prior research in different settings demonstrated that lack of knowledge or training decreased healthcare workers’ comfort with buprenorphine/naloxone initiation, and this has also been identified in the ED physician population [9–11, 13–15, 19–22]. Previous studies among healthcare workers identified logistical challenges as significant barriers to prescribing medications for OUD. In this vein, healthcare workers in our study identified lack of access to follow-up pathways as a barrier, similar to prior research [19–22]. A prominent logistic barrier cited by ED physicians is time, which is consistent with our findings [19–21]. Healthcare workers discussed both the time required to complete buprenorphine/naloxone interventions with individual patients, and limited time available in the context of high patient volumes in the ED. Based on data from our feasibility study, mean time to counsel patients was 12 minutes (SD 4). This would be an additive healthcare provider task above the time required for the patient’s general assessment. This time is comparable to what would be necessary for a de novo emergency physician patient assessment and would limit providers’ ability to tend to one additional patient.

The healthcare workers in our study did not commonly report stigma as a barrier, yet stigma toward patients with substance use disorder is a well-known barrier to providing buprenorphine/naloxone [9–14]. In prior outpatient studies a way that stigma was identified was when healthcare providers stated patients with OUD were difficult to treat and/or they did not want to attract patients with OUD to their practice. Prior to study commencement we attempted to minimize stigma towards people with OUD. Pharmacists attended a live session from a mother advocate from the organization, ‘Moms Stop the Harm’ [23]. Healthcare workers in the ED do not have the ability to restrict which patients they see and all the patients in this study would have been treated by the ED for their chief complaint regardless of the initiation of buprenorphine/naloxone, and this may have minimized identified stigma.

### Strengths and limitations

Our study is strengthened by our success in engaging multidisciplinary healthcare worker groups to gain a broad understanding of facilitators and barriers to ED buprenorphine/naloxone provision, which adds a novel and important perspective to the literature in this field. We meaningfully engaged ED healthcare worker groups because interdisciplinary collaboration was a foundational aspect of the planning and implementation of our ED take-home buprenorphine/naloxone program from the outset, which allowed us to build trust and goodwill. Furthermore, our ability to meaningfully respond to healthcare workers’ feedback enhanced workers’ comfort in sharing openly with our study team. As an example, information from our qualitative work informed improvements of our training programs, study supports, and patient follow-up mechanisms through existing partnerships of our study team with community outreach workers. Although our follow-up mechanisms were not perfect and were identified as an area of potential improvement, the existence of low-barrier options is a support not available to all EDs and hence a strength upon which we can build. Additionally, we engaged people with lived and living experience of opioid use throughout study design and implementation. This allowed us to integrate important patient feedback from the outset to enhance the successful uptake of our program.

The primary limitation of this study is potential selection biases introduced by: 1) our inclusion criteria (e.g., healthcare workers who had cared for patients provided buprenorphine/naloxone), and 2) by self-selection of healthcare providers who consented to interviews. By selecting healthcare workers who had some experience in providing care to a patient who was started on buprenorphine/naloxone, we may have selected for healthcare workers who had more positive perceptions of the care of patients with substance use disorder care in the ED. In addition to broad screening by our research assistants, our feasibility study relied on healthcare worker referrals to identify eligible patients. Therefore, it is possible that healthcare workers who had positive perceptions of initiating treatment for substance use disorder from EDs were more likely to identify patients for enrollment. Those who felt more positively toward take-home buprenorphine/naloxone and harm reduction in general may have been more likely to participate in the qualitative study and less likely to report negative or critical feedback. Another potential limitation is the design of our buprenorphine/naloxone intervention and study processes: as this was a feasibility study, we had not yet streamlined our processes to optimize efficiency of processes (e.g., timing of consenting, counseling, enrollment tasks). Therefore, identified barriers may be related to inefficiencies that we have addressed in ongoing iterations of our program. Additionally, our study was limited a single, urban, tertiary care ED, and describes experiences of providers who had initiated buprenorphine/naloxone in a small number of patients.

### Clinical implications

Our qualitative work with multidisciplinary ED healthcare workers reinforced that healthcare workers largely consider buprenorphine/naloxone provision to be within the scope of ED healthcare workers responsibility. The ED is also a place that can be accessed at any time of day by individuals with substance use disorders, at the point in time when they are ready for treatment. Focusing on making the ED a more favorable place to stay (e.g., providing food), treating patient symptoms (e.g., opioid withdrawal), and prioritizing the timing of conversations around substance use disorder may minimize patients leaving prior to receiving take-home buprenorphine/naloxone. Identifying confidential places in the ED to have conversations around substance use disorder prior to providing take-home buprenorphine naloxone would minimize another identified barrier. Interventions must also seek to leverage and strengthen organizational and interpersonal factors to facilitate buprenorphine/naloxone provision. Strong training programs on buprenorphine/naloxone are facilitive and may also lead to more expedited care, which may also minimize patients leaving prior to receiving buprenorphine/naloxone. Healthcare workers identified that, despite challenges of operating in a busy ED environment, distributing the workload and providing training across multiple healthcare team members mitigated the burden on any single healthcare worker and allowed the ED team to manage the complexities of addiction care even at times of increased volume.

### Research implications

Further research should focus on perceptions of healthcare workers stratified along a spectrum of experience levels in initiating buprenorphine/naloxone. Future research should seek to identify facilitators and barriers to buprenorphine/naloxone provision at sites with different geographic distributions (e.g., rural/suburban/urban), and different levels of resource supports (e.g., addictions consultation services; follow-up pathways; ED harm reduction supplies). Given that this research focused on healthcare providers’ perspectives of providing take-home buprenorphine/naloxone, future research should focus on elaborating patient perspectives on facilitators and barriers to ED buprenorphine/naloxone programs.

## Conclusion

Canada is in the midst of an epidemic of opioid overdose-related deaths, and our healthcare system must increase ways for individuals with OUD to access addiction care when they are ready. The healthcare workers we interviewed recognized the important opportunity afforded by ED visits to identify patients who could benefit from buprenorphine/naloxone, and to improve access to this treatment. This qualitative study describes key facilitators and barriers to implementing successful ED buprenorphine/naloxone programs from multidisciplinary healthcare providers’ perspectives. Institutional supports should address the factors we have identified (e.g., provide concerted education, address logistical challenges, and understand unique space and time constraints) to increase ease of implementation and mitigate anticipated barriers.

To support broader implementation, training programs, greater clinical exposure to buprenorphine/naloxone prescribing (e.g., mentorship programs), dedicated follow-up pathways, and human resource support may alleviate the burden of ED factors that are difficult to change, such as volume and space availability. Integrating a buprenorphine/naloxone program into ED care is a viable response to the opioid overdose epidemic with education of healthcare workers and organizational supports.

### Supplementary Information

**Additional file 1: Appendix 1.** Induction protocols and counselling handouts.

**Additional file 2: Appendix 2.** Discussion guide for healthcare worker interviews.

**Additional file 3: Appendix 3.** Consolidated criteria for reporting qualitative studies (COREQ): 32-item checklist.

**Additional file 4: Appendix 4.** Additional study methods.

## Data Availability

The datasets generated and/or analyzed during the current study are not publicly available because consent was not obtained for individual transcripts to be publicly available, but are available from the corresponding author (Katherin Badke katherin.badke@vch.ca) on reasonable request.
