# Supplementary material for: Healthcare provider perspectives on emergency department-initiated buprenorphine/naloxone: a qualitative study
Source: BMC Health Serv Res. 2024 Feb 15;24:211. doi: 10.1186/s12913-023-10271-7 (PMC10870432; doi:10.1186/s12913-023-10271-7)
Supplement: Supplementary file 1 — Additional file 1: Appendix 1. Induction protocols and counselling handouts. [file 12913_2023_10271_MOESM1_ESM.docx]

**Appendix 1: Induction protocols**

**Buprenorphine/naloxone standard dose**

Three-day take-home buprenorphine/naloxone standard-dosing package

Patients were counselled to start after their emergency department visit once they were experiencing moderate opioid withdrawal and to avoid opioid use during induction.

Initial dose was 2/0.5 mg sublingual, which could be repeated hourly to a maximum of 12/3 mg sublingual in 24 hours.

For days two and three, patients took the total dose achieved on day one (target 12/3 mg).

**Buprenorphine/naloxone microdose**

Six-day take-home buprenorphine/naloxone microdosing bubble package using buprenorphine/naloxone 2/0.5 mg sublingual tablets.

Patients were counseled that they could initiate the first dose at any time that was convenient for them (including in the emergency department, if preferred) and to continue concurrent illicit opioid use during induction, with no requirement for opioid withdrawal.

Day one

Buprenorphine/naloxone 0.5/0.125 mg sublingual twice daily

Day two

Buprenorphine/naloxone 1/0.25 mg sublingual twice daily

Day three

Buprenorphine/naloxone 2/0.5 mg sublingual twice daily

Day four

Buprenorphine/naloxone 3/0.75 mg sublingual twice daily

Day five

Buprenorphine/naloxone 4/1 mg sublingual twice daily

Day six

Buprenorphine/naloxone 12/3 mg sublingual once daily
