# Supplementary material for: Healthcare provider perspectives on emergency department-initiated buprenorphine/naloxone: a qualitative study
Source: BMC Health Serv Res. 2024 Feb 15;24:211. doi: 10.1186/s12913-023-10271-7 (PMC10870432; doi:10.1186/s12913-023-10271-7)
Supplement: Supplementary file 2 — Additional file 2: Appendix 2. Discussion guide for healthcare worker interviews. [file 12913_2023_10271_MOESM2_ESM.docx]

**Appendix 2: Discussion guide for healthcare worker interviews**

**Welcome and introductions**

**Guidelines**

We will be asking you questions about buprenorphine/naloxone

No right or wrong answers; just different points of view

Session will be recorded; reminder of confidentiality

For focus groups: listen respectfully; only one person speaks at a time

Ensure cellphones are on vibrate

**Verbal consent script**

**Reimbursement:** You will be given a $5 coffee shop gift card to reimburse you for your time today. You will receive the gift card regardless of if you complete or withdraw from the study.

**Introduce topic:** We’re talking about outpatient buprenorphine/naloxone initiated from the emergency department

Facilitator to hand out form asking years worked in profession and number of buprenorphine/naloxone starts they have been involved in and regimen provided

If they can’t come up with an exact number then ask them for the following range

0-5 starts

6-10 starts

10-19 starts

20+ starts

**Questions**

1. *Experience with buprenorphine/naloxone*
2. Describe your experience with buprenorphine/naloxone prior to being involved in the care of one or more of our study participants.
3. What kind of training have you received on buprenorphine/naloxone
   1. [prompt] On the job training? In school?
4. Would you have wanted any additional training prior to being involved in emergency department buprenorphine/naloxone initiation?
5. What are your views about or understanding of buprenorphine/naloxone and opioid agonist therapy for opioid use disorder? [microdose vs. standard dose]
6. What do you feel your role is regarding starting buprenorphine/naloxone in the emergency department?
   1. What about the role of the emergency department in starting people on buprenorphine/naloxone?

In this next set of questions, we want to explore your experience [starting / suggesting starting] buprenorphine/naloxone.

1. What was your process for identifying patients that might be eligible for buprenorphine/naloxone?
2. [If physician / pharmacist / nurse] Describe your experience with the process and workflow of getting buprenorphine/naloxone.
   1. How did it impact your usual workflow?
   2. Did you understand the process?
   3. Did you encounter any barriers?
   4. Do you have any thoughts about the pre-printed order?
3. [If social worker] Describe your workflow around referring a patient to be assessed for take-home buprenorphine/naloxone.
   1. How did it impact your usual workflow?
   2. Did you understand the process?
   3. Did you encounter any barriers?
4. Describe your experience caring for a patient started on outpatient buprenorphine/naloxone from the emergency department.
   1. [prompt] Did you encounter any specific challenges with this patient population?
5. [If physician / pharmacist / nurse] Describe your experience counselling patients on buprenorphine/naloxone.
6. Did this process have any impact on patient flow in the emergency department? How so?
7. *Facilitating factors*
8. Based on your experiences, what made it easy or helped to initiate buprenorphine/naloxone in a patient in the emergency department?
9. Can you think of anything else that could facilitate this process?
10. *Challenges*
11. [If physician / pharmacist / nurse] What challenges did you experience in providing buprenorphine/naloxone to emergency department patients?
12. [If social worker] What challenges did you experience in referring emergency department patients for buprenorphine/naloxone assessment?
13. Can you think of any additional potential barriers to providing buprenorphine/naloxone to emergency department patients?
14. What suggestions do you have to improve the provision of buprenorphine/naloxone in the emergency department?
15. *Suggestions/feedback*
16. How should the emergency department be identifying people with opioid use disorder?
    1. [prompt] Who should be responsible? At what point during the patient’s health care encounter?
17. How comfortable would you be [starting / suggesting starting] buprenorphine/naloxone without the current study support? *training?
18. We are interested in studying the effect of two dosing regimens through a comparative trial or other effectiveness study. What do you think we could do to make a randomized controlled trial of buprenorphine/naloxone starts in the emergency department most successful?
19. *Open the Floor*
20. Any additional comments?
21. *Additional questions based on prior focus groups*

Conclusion

Wrap up and Debrief: Re-affirmation of confidentiality.

Provision of contact information for study team.

If you have any additional comments, you can contact the study team. After we have reviewed the notes from this focus group, we may contact you/members of this focus group to ensure that we properly understand your responses.
