# Supplementary material for: Healthcare provider perspectives on emergency department-initiated buprenorphine/naloxone: a qualitative study
Source: BMC Health Serv Res. 2024 Feb 15;24:211. doi: 10.1186/s12913-023-10271-7 (PMC10870432; doi:10.1186/s12913-023-10271-7)
Supplement: Supplementary file 4 — Additional file 4: Appendix 4. Additional study methods. [file 12913_2023_10271_MOESM4_ESM.docx]

**Appendix 4: Additional study methods**

Our team conducted a descriptive qualitative study at an urban tertiary hospital. Study participants were healthcare workers who provided care to study participants who were enrolled in phase 1 and phase 2 of a previously published feasibility study [1]. In phase 1 of the feasibility study, our focus was on the logistics of identifying eligible patients and initiating intervention in the emergency department. In phase 1, we implemented two interventions sequentially (aiming for 15 patients in each block). The first block of patients was offered standard dose buprenorphine/naloxone and the second block was offered microdose buprenorphine/naloxone. In phase 2, patients were randomized (1:1) to either standard dosing or microdosing buprenorphine/naloxone.

**Healthcare worker education**

We anticipated that pharmacists would do most of the counseling, so their education plan was the most thorough. All pharmacists completed two online training modules provided by the health authority, one on buprenorphine/naloxone, and one on naloxone. Pharmacists also received four separate 45-minute lunchtime learning sessions on addiction care, standard dose buprenorphine/naloxone, microdose buprenorphine/naloxone, and take-home naloxone counseling. Prior to study commencement we attempted to minimize stigma towards people with opioid use disorder. Pharmacists attended a live session from a mother advocate from the organization, ‘Moms Stop the Harm’ [2]. Pharmacists who did not attend any session watched the recording and then discussed the material with the emergency department clinical pharmacy specialist (KB). Interested pharmacists participated in a 45-minute group session on the spirit of motivational interviewing from a motivational interview trainer.

We experienced greater challenges arranging training for nurses, social workers and physicians due to scheduling variability, however significant interdisciplinary education was offered. A buprenorphine/naloxone information session occurred at the nurse education day and at physician rounds. The health authority online training modules on buprenorphine/naloxone and naloxone were available to all healthcare workers. Social media was used to upload information for staff, and study team members brought an education board through the emergency department on various days to target a wide variety of staff. Pre-preprinted orders, with buprenorphine/naloxone contraindications listed, were created to make ordering buprenorphine/naloxone an easier process for the healthcare team.

**Patients**

Trained research assistants screened the emergency department census for patients who were 18 years or older using a predetermined list of presenting complaints. Staﬀ members in the emergency department could also identify potential patients. Patients were enrolled during the hours of 0800-2300 Monday to Friday and 1300 to 2100 Saturday, Sunday and statutory holidays. The research assistants approached patients who did not have active opioid agonist therapy on PharmaNet (provincial registry of medications dispensed from community pharmacies) and asked about non-medical opioid use in the previous 30 days. If the patients had non-medical opioid use in the previous 30 days, the research assistants screened them for eligibility for buprenorphine/naloxone treatment. This screening procedure consisted of the Rapid Opioid Dependency Scale, a validated screening tool for opioid dependence [3]. Patients who were eligible for initiation of buprenorphine/naloxone were offered participation in the feasibility study. Research assistants reviewed a written consent form with patients prior to enrollment, which the participants signed.

**Healthcare worker recruitment**

Eligible healthcare workers were those who cared for patients in phase 1 and phase 2 of the feasibility study. As the phases were consecutive, healthcare workers may have provided care to patients in phase 1, phase 2, or both phases of the study.

**Interviews and focus groups methods**

The research team developed a semi-structured interview guide. We developed the discussion guide based on key themes in the literature, which the study team (pharmacist, nurse, social worker, physician, and qualitative researcher) iteratively reviewed to provide different disciplinary lenses. The qualitative researcher conducted semi-structured interviews among emergency physicians, nurses, pharmacists, and social workers until we reached data saturation or there were no additional interested/consenting participants. We achieved data saturation for the physicians, nurses and pharmacists. Due to the small number of social workers, we conducted interviews until there were no additional consenting participants. Initially, we conducted interviews and focus groups in-person at a research office in Vancouver General Hospital, but we shifted to telephone-based interviews due to the COVID-19 pandemic restrictions.

Interviews were supplemented with field notes and audio recorded. We assigned interview participants a unique study number. The healthcare interviews and focus groups lasted 14 to 120 minutes. Research assistants transcribed the interviews verbatim. After transcription, we destroyed all audio files and removed identifying information from the transcriptions. We stored transcribed files on a password-protected file on a secure network, as approved by ethics.

**References**

1. Moe J, Badke K, Pratt M, Cho RY, Azar P, Flemming H, et al. Microdosing and standard-dosing take-home buprenorphine from the emergency department: A feasibility study. J Am Coll Emerg Physicians Open. 2020 Oct 20;1(6):1712-1722. doi: 10.1002/emp2.12289.
2. Moms Stop the Harm. MOMS STOP THE HARM [Internet]. 2022. [cited 2023 October 23]. Available from: https:// www. momss topth eharm. com/
3. Wickersham JA, Azar MM, Cannon CM, Altice FL, Springer SA. Validation of a brief measure of opioid dependence. J Correct Health Care. 2015;21(1):12-26.
